# SARS-CoV-2 prevalence in an asymptomatic cancer cohort - results and consequences for clinical routine

**DOI:** 10.1186/s13014-020-01609-0

**Published:** 2020-07-09

**Authors:** Sebastian Marschner, Stefanie Corradini, Josefine Rauch, Richard Zacharias, Ana Sujic, Julia Mayerle, Raluca Petru, Béatrice Grabein, Oliver T. Keppler, Edwin Boelke, Claus Belka, Maximilian Niyazi

**Affiliations:** 1 Department of Radiation Oncology, University Hospital, LMU Munich, Munich, Germany; 2 German Cancer Consortium (DKTK), Partner Site Munich, Munich, Germany; 3 Department of Internal Medicine II, University Hospital, LMU Munich, Munich, Germany; 4 Institute and Outpatient Clinic for Occupational, Social and Environmental Medicine, WHO Collaborating Centre for Occupational Health, LMU University Hospital, Munich, Germany; 5 Occupational Medical Centre, LMU University Hospital, Munich, Germany; 6 Department of Clinical microbiology & hospital hygiene, University Hospital, LMU Munich, Munich, Germany; 7 Max von Pettenkofer Institute, Virology, Faculty of Medicine, LMU Munich, 80336 Munich, Germany; 8 Department of Radiation Oncology, Heinrich Heine University of Düsseldorf, Düsseldorf, Germany

**Keywords:** SARS-CoV-2, COVID-19, Coronavirus, Radiation oncology, Radiotherapy, Screening, PCR

## Abstract

**Background:**

Starting in December 2019, the current pandemic caused by the severe acute respiratory syndrome coronavirus 2 (SARS-CoV-2) confronts the world with an unprecedented challenge. With no vaccine or drug being currently available to control the pandemic spread, prevention and PCR (Polymerase chain reaction) testing becomes a crucial pillar of medical systems. Aim of the present study was to report on the first results of the measures taken in a large German Department of Radiation Oncology, including PCR testing of asymptomatic cancer patients.

**Methods:**

Pandemic-adapted hygiene regulations and prevention measures for patients and staff were implemented. A visiting ban on both wards was implemented from the beginning and medical staff and patients were required to wear face masks at all times. The waiting rooms were rearranged to ensure distance between patients of at least 1.5 m. Clinical follow up was mainly done by telephone and all patients had to complete a questionnaire regarding symptoms and contacts with COVID-19 patients before entering our department. Educational documents were created for patients to raise awareness of symptoms and avoidance strategies for interactions with other people. Indications for therapy and fractionation schemes were adapted when possible. In a subsequent step, all new asymptomatic patients were tested via nasopharyngeal swab at our screening station shortly before their simulation CT.

**Results:**

All these measures and implementations have been well accepted semiquantitatively measured by the consent received from patients and staff. Regarding the PCR testing, only 1 out of 139 asymptomatic patients of our cohort so far tested positive for SARS-CoV-2, reflecting a prevalence of 0.72% in this cancer patient population. Up to this point no staff members was tested positive. The start of the treatment for the PCR-positive patient was deferred for 2 weeks.

**Conclusion:**

Due to the pandemic-adapted implementations, our department seems well prepared during this crisis. The initial screening helps to identify asymptomatic COVID-19 patients in order to protect other patients and our staff from infection and the observed PCR prevalence is in line with comparable studies. A regular PCR testing (e.g. twice a week) of all patients and staff would in principle be desirable but is limited due to testing capacities at present.

## Background

The world has changed since the outbreak of the novel severe acute respiratory syndrome coronavirus 2 (SARS-CoV-2) causing coronavirus disease 2019 (COVID-19). Starting in December 2019 with a cluster of severe pneumonia in China, it has become a worldwide pandemic. As in two preceding outbreaks of coronavirus disease in the past 18 years — SARS (2002 and 2003) and Middle East respiratory syndrome (MERS) (2012 to the present) —SARS-CoV-2 has posed critical challenges for all public health care systems and medical communities [1, 2]. With more than 4.0 million confirmed cases and 280,000 deaths until May 11th 2020 worldwide, the virus has affected almost every country and society [3–7].

The virus itself belongs to the family of *Coronaviridae* [8]. It is transmitted by droplet infection and can spread very quickly compared to previously known viruses. The incubation period can last up to 14 days with an average time of 4–5 days until the appearance of initial symptoms. These are usually cold-like symptoms such as cough, fever and fatigue [9, 10]. However, in some cases the virus can cause viral pneumonia (COVID-19) with additional extrapulmonary manifestations. These complications are associated with a high overall inflammatory burden and vascular inflammation [11].

A considerable proportion of infected patients is asymptomatic. These are mainly younger people who, as asymptomatic carriers, can silently infect other people and thus accelerate the pandemic [12, 13]. First analyses of previously infected and deceased patients in China and the US show that the case fatality rate was highest among elderly persons and for patients with comorbidities, such as heart disease, arterial hypertension, diabetes or chronic lung disease. Accounting for differences in age and prevalence of underlying comorbidity, COVID-19 associated mortality in the United States was similar compared to China [10, 14–22].

Studies have confirmed that COVID-19 patients with cancer had poorer outcomes potentially due to immunosuppression during cancer therapy. Additional high incidence of comorbidities in these patients increases the probability of an unfavorable course [15–17]. Thus, almost every patient in an oncological department is considered to be at risk for a severe SARS-CoV-2 infection. Therefore, physicians should pay more attention to patients with cancer in case of rapid deterioration [5, 10, 23–26].

More than 50% of all cancer patients will be treated with radiotherapy at some stage of their disease, making Radiation Oncology (RO) a key discipline in oncology [9]. An infection with SARS-CoV-2 during treatment would lead to a delay, interruption or even discontinuation of cancer therapy and thus influence the oncological outcome detrimentally. Subsequently, if radiotherapy staff, especially RTTs, gets infected, risk of super-spreading is increased (due to a high number of close patient contacts per day), and patient treatment is at risk, if the number of cases is critical concerning the patient workflow [4].

In order to maintain optimal patient care, it is crucial to develop an appropriate hygiene, surveillance and screening concept and to establish clear protocols in case of infection of patients or staff [3].

As radiation oncology department in the second largest university hospital in Germany, we want to report on the implementation, the progress and first results of our hygiene, prevention and screening protocols in order to aid other departments that are still in their earlier stages.

## Patients & methods

### Prevention

Since there is no specific therapy or vaccination against the virus at present, the main focus is currently on preventive measures to minimize the risk of infection for patients and medical staff. As it can be observed in other countries such as Italy or Spain, infected medical staff can be inconspicuous, but also a potential source of infection, which is transmitted during every patient contact [27].

Overall, medical staff, patient and device hygiene are crucial measures on wards, radiotherapy units, offices as well as all other publicly accessible areas [9].

In the daily clinical routine of a radiation oncology department with two campuses, approximately 180 patients per day and 2 wards (capacity for 50 patients), frequent patient contact is inevitable. While a distance of 1.5 m can be kept during an outpatient consultation, this is impossible during e.g. a physical examination or brachytherapy. Therefore, it is necessary to minimize the risk of infection for staff and patients while maintaining the best possible care at the same time. With initially limited test capacities, comprehensive screening has not been available and conservative preventive measures had to be taken. This is why careful prescreening by telephone and at presentation of patients became the most important elements in our department at the beginning, alongside with hygiene measures and social distancing.

### General implementations

All patients and staff members were instructed on proper hygiene during the medical consultations and chart rounds according to the adapted World Health Organization (WHO), Robert Koch Institute (RKI) and internal hospital guidelines.

Initially, medical staff was required to wear surgical face masks for all patient contacts, and patients had to wear face masks during medical visits and physical examinations. Now, as the pandemic progressed, all patients and hospital staff must wear face masks at all times. FFP2/3 masks are only used if a patient was tested positive or the suspicion of an infection exists. These measures have already led to a decrease of needed tests at the hospitals main staff screening station.

Novel documents based on the specifications of DEGRO and other oncological umbrella organizations were developed for patients, distributed and discussed to raise awareness of symptoms, avoidance strategies during activities of daily living and important details on interactions with other people in the household (see supplemental documents 2 and 3) [28].

The waiting rooms were converted, so that each patient had at least 1.5 m distance to the next patient. Patients were asked to attend our department right on time before their scheduled appointment to avoid large accumulations of patients in the waiting room. Accompanying persons were allowed for translation or medical reasons only.

At the Linacs, elderly and frail patients, patients with immunosuppressive therapies or patients receiving thoracic radiotherapy were irradiated in the morning. If present, patients with mild symptoms were irradiated at a dedicated Linac with spatiotemporal separation. Treatments of PCR proven asymptomatic SARS-CoV-2 carriers is under debate and has not been carried out so far – these cases would require a high security level to guarantee a maximum protection for staff members involved.

In the hypothetical case of a large number of infected patients, one Linac would be selected with a separate waiting area to prevent contacts to other patients. As our department is not accessible via a separate entrance, treatment of SARS-CoV-2-positive patients would be carried out at this unit at the end of the day. This would have ensured a regulated and sealed-off workflow even with a large number of infected patients requiring radiotherapy. Fortunately, there was no need to implement this workflow so far.

### Outpatients

The indications of already scheduled outpatient visits for consultation were strictly reconsidered. Patients with benign, less aggressive diseases, or questionable indications were contacted by phone to discuss further treatment and to avoid unnecessary visits. Curative patients with an indication for adjuvant therapy were postponed as long as possible, according to the available literature. For indications such as prostate cancer for example, antiandrogen therapy (if indicated) was started as a bridging therapy and the beginning of radiotherapy was postponed.

Depending on the cancer entity, hypofractionation was a preferred choice when available literature allowed it [9]. However, all adaptations made during treatment planning were intended not to affect the patient outcome. These initial steps were necessary to reduce the number of patients, reorganize the departmental structure and at the same time provide a tandem staff to maintain radiotherapy in urgent indications, and to cope with challenges such as infected staff members or the deployment of staff to other hospital units.

Each patient who had been approved for therapy was contacted beforehand by telephone to answer the standardized questionnaire (see supplemental document 1). If an infection was suspected, a nasal swab test was indicated via the clinic’s screening station at the emergency room and patients started therapy only if the test result was negative. At each further appointment, all patients had to update their answers to this questionnaire. This led to a consistent monitoring of all patients and the possibility to react immediately if symptoms appeared. The result was confirmed by signature on a daily basis and remained in the patient chart.

Follow-up was carried out almost entirely by telephone and patients were only invited for a personal consultation when deemed necessary. Patients were asked to send diagnostic imaging or medical reports and were contacted after reviewing all documents.

### Inpatients

The first step was a screening of existing inpatients and increased frequency of body temperature measurements up to 4 times per day. Patients who were scheduled for a transfer from other wards were only admitted to our ward if they had a negative SARS-CoV-2 test result shortly before transfer. External patients who were scheduled for admission the next day were contacted by telephone the evening before their arrival, using our questionnaire, e.g. questions regarding travel to so-called “risk areas” or contact with SARS-CoV-2-positive people. Access to the ward was only granted, if their medical and virus-related history was inconspicuous.

We tried to adopt a single-room solution for our inpatients and a complete visitor ban had been established right from the beginning of the pandemic. Existing hygiene concepts were intensified and discussed extensively with all staff members. Nurses were assigned to specific patients and allocation changes were avoided. The early introduction of telephone screening and face masks has so far prevented any infection of staff.

### Screening

Due to the initial lack of test kits, screening was only available in symptomatic patients. This implicated a relevant risk that patients and medical staff could silently infect each other, which could quickly lead to a health care problem within the department.

Since almost every patient in the radiation oncology departments belongs to the often mentioned “group at risk” and the capacities of test kit were available, we then decided, in agreement with the hospital’s “Corona task force”, to screen all radiotherapy patients in our department shortly before the start of therapy.

Our motivation was that a positive test result represents a therapy-relevant decision and, if undetected, could cause serious adverse events. In addition, every patient comes into contact with many staff members and other patients during the course of their therapy, which could last up to 35 fractions or more, and therefore protection of employees is of crucial importance.

Three of our staff members were trained at the hospitals main staff screening station and instructed on hygienic concepts and internal clinical procedures like the process of dispatching test kits. The department’s day clinic on one of our wards was converted into a screening station according to the current hygiene standards and the adjacent patient room was converted into a waiting area with sufficient distance between chairs. This ensured minimizing contacts to other patients on the ward. Ten-minute slots were established, and patients were asked to keep their time frame to avoid larger groups in the waiting room and insure the hygienic disinfection of surfaces in the test room after every patient. Testing was carried out on 2 days per week for 5 h with about 25 patients per day. The results were available 1 day later and 1–2 days prior to their simulation CT. With increasing demand, a third screening day was introduced after 2 weeks.

On the screening day itself, two of the trained employees were assigned to the test station. One was responsible for patient guidance and preparation of the test items, the second person was responsible for testing. Testing personnel wore full personal protective equipment including a disposable fluid repellent coverall, FFP2 mask and eye protection shield. Since only one person performed the tests in a specially assigned room with restricted contact to the rest of the department ensured hygienic standards and avoidance of unnecessary consumption of protective equipment. Only a change of gloves and hand disinfection was necessary between two individual patients if no other visible contamination occurred. After their test, all outpatients had to go home again, while the inpatients were admitted to a single room on the ward until the results of the test were available.

For testing, eSwab (Copan Italia SpA, Italy) test kits with a liquid amies preservation medium were used and the smear was routinely taken by a nasopharyngeal swab. If access through the nasal cavity was not feasible, the smear was taken orally using an oropharyngeal swab with the same test kit. The test was carried out by the in-house microbiological institute via PCR (SARS-CoV-2-RNA N-Gen 1) within 1 day. PCR starts with laboratory conversion of viral genomic RNA into DNA by RNA-dependent DNA polymerase (reverse transcriptase). Afterwards, small DNA sequence primers designed to specifically recognize complementary sequences on the RNA viral genome and the reverse transcriptase generate a short complementary DNA copy (cDNA) of the viral RNA. This process is repeated for multiple cycles until the viral cDNA can be detected, usually by a fluorescent or electrical signal [29–31].

Patients with negative results were informed at the day of simulation CT. In the case of a positive result, the patient was first informed in order to discuss necessary protective measures for themselves and for other people in the household. The patient was then also reported to the public health department. Afterwards, the responsible senior physician was consulted and further therapeutic procedures were discussed internally with regard to a delay or omission of therapy considering the age, diagnosis, indication and clinical performance status of the patient.

This study complies with the declaration of Helsinki, Good Clinical Practice (GCP) and Good Epidemiological Practice (GEP). The data acquisition and analysis were in accordance with Bavarian hospital law (Art.27 Abs. 4 BayKrG). This work did not require written patient consent.

## Results

All asymptomatic patients were tested consecutively after they gave their informed consent but before starting radiotherapy. From April 17th to May 8th, 2020 overall 139 patients were tested: 74 female and 65 male patients. The prevalence of SARS-CoV-2 in our cohort was 0.72%. A full overview of the tested patients with their indication is listed in Table 1.

**Table 1:** Patient characteristics

		Number (***n*** = 139)			Number (***n*** = 139)
**Result**	Positive	1 (0.72%)	**Radiation** **Technique**	3D-CRT	30 (21.6%)
	Negative	138 (99.3%)		VMAT	83 (59.7%)
				SRS/SRT/SBRT	18 (13.0%)
**Age**	< 40	9 (6.5%)		Brachytherapy	5 (3.6%)
	40–49	15 (10.8%)		Others	3 (2.2%)
	50–59	32 (23.0%)			
	60–69	25 (18.0%)	**Entity**	Brain	13 (9.4%)
	> 70	58 (41.7%)		Head & Neck	11 (8.0%)
				Breast	36 (25.9%)
**Gender**	Male	65 (46.8%)		Gynaecological	12 (8.6%)
	Female	74 (53.2%)		Lung	24 (17.3%)
				Gastrointestinal	12 (8.6%)
				Bladder	2 (1.4%)
**Intention**	Curative	95 (68.3%)		Prostate	17 (12.2%)
	adjuvant	59 (42.4%)		Lymphoma	3 (2.2%)
	definitive	36 (25.9%)		Sarcoma	4 (2.9%)
	Palliative	44 (31.7%)		Melanoma	4 (2.9%)
				Others	6 (4.3%)

*3D-CRT* 3D conformal radiation therapy, *VMAT* Volumetric-modulated arc therapy, *SRS* Stereotactic radiosurgery, *SRT* Stereotactic radiotherapy, *SBRT* Stereotactic body radiation therapy

Only one male patient (0.72%) has been tested positive for SARS-CoV-2, all others were negative (99.28%). The patient’s history of infectious symptoms was asymptomatic before testing. In a telephone call 1 day after the test, he still had no symptoms but his daughter living with him already had a slight fever for 2 days. However, she had not been tested for SARS-CoV-2 at this point.

With an age of 81 years, the diagnosis of recurrent non small cell lung cancer and a history of diabetes, heart disease, long known dyspnea at rest (NYHA III) and a smoking history of 200py, this patient was a high-risk patient.

In the subsequent case discussion, the risk of a deterioration of the general condition due to chemoradiotherapy and the resulting increased risk of COVID-19 motivated us to postpone his treatment for 2–3 weeks. Therefore, a home quarantine was ordered for 2 weeks and subsequent re-testing afterwards. If, in the meantime, a worsening of the symptoms became apparent, an immediate presentation in our emergency department was recommended.

During his home quarantine, no symptoms occurred and 3 weeks later, the patient tested negative twice. In the following planning CT no infiltrates or other SARS-CoV-2 related anomalies could be seen and therefore definitive chemoradiation has been initiated. After therapy completion, no abnormalities were observed and the therapy was well tolerated. A deterioration of the long known dyspnea at rest could not be determined.

## Discussion

Since the outbreak of the SARS-CoV-2 virus in Wuhan in December 2019 and its worldwide spread, multiple studies have attempted to discover more details about the virus, such as incubation times, transmission, virulence, reproduction times or population infestation rates. These figures are needed to better estimate the risk of infection for patients and medical staff. All this will help to remove current uncertainties and provide political decision-makers and health care systems with accurate figures as a solid data base for further prevention measures.

One of the crucial parameters is the proportion of asymptomatic carriers. An initial shortage of test kits resulted in exclusive testing of symptomatic patients. However, this prevented a final statement about the actual infestation rate. As known from other viruses, the proportion of asymptomatic carriers can range from 8% for measles, 30–35% for norovirus and up to 90–95% for polio [32–34]. Recent studies show that about 20–35% of those infected with SARS-CoV-2 are asymptomatic carriers [34, 35]. In measles and norovirus, risk and time of transmission in the asymptomatic stage have already been characterized, but we know very little about asymptomatic COVID-19 patients and how long they can transmit SARS-CoV-2. Since there are currently only estimates of untested deaths or asymptomatic carriers, overall mortality may not be as accurate: it is estimated to be between 1 and 8% depending on the region or population tested. In the heavily affected small German town of Heinsberg, scientists reported a prevalence of 15% and a mortality rate of only 0.8% [36]. Comparing mortality, this is lower than in two previous epidemics (SARS and MERS) with 10% for SARS and 34% for MERS [37]. However, different risk groups of COVID-19 patients have shown higher mortality rates, e.g. Liu et al. recorded mortality rates of up to 15% in patients > 80 years, about 8% in patients aged 70 to 80 years and 7.8% for cancer patients [26, 38]. Due to the rapid spread of SARS-CoV-2, the current pandemic has resulted in a significantly higher death count, which has caused shortcomings in the health care system in many countries.

In clinical routine, asymptomatic carriers are the most challenging ones. Firstly, because the risk of a severe course of COVID-19 increases as the treatment with chemoradiotherapy progresses, and secondly because they can infect other patients or medical staff silently.

All this led to our decision to test all of our new patients before their initiation of radiotherapy at our department. The optimal time point for testing must be carefully chosen as the effectiveness of RT-PCR for detection of SARS-CoV-2 has been shown to be largely influenced by the quantity, type and timing [39, 40]. If the test is done too early, its reliability is limited, as there is a risk that the patient could become infected in the meantime. The later the test is performed, the more staff will get into contact with patients of unknown infectious status.

A single test can be false negative even though the infection already exists or because it has just started. False negative results could also emerge due to the quality of the swab by unexperienced staff. This risk was minimized by training our staff at the hospitals main staff screening station for 1 day under supervision of experienced personnel.

An additional limitation is the reported sensitivity with range from 80 and 54% of the PCR for nasopharyngeal swab [40, 41]. This limitation could only be improved by repeated testing. This would increase the validity of respective results, but is currently infeasible due to the shortage of test kits. And even if so, there is no existing policy on which intervals would be appropriate for retesting.

In our situation with a high number of daily and many patients with multiple comorbidities, we decided to test once 2–3 days before the simulation CT for outpatients or at the arrival on the ward for inpatients, if they were not tested elsewhere. This guaranteed a test result immediately before the planning CT and the relative safety of the respective staff. Afterwards and especially during therapy, the patients were in our department on a daily base and worsening or even the beginning of symptoms would be noticed by the staff by using the standardized questionnaires. In this case, a patient retest would be initiated. Additional patient guidelines, which were prepared according to the existing regulations of RKI and WHO, trained the patients as far as possible in order to be able to implement preventive measures and to recognize the first symptoms themselves. Since most of our patients are very conscientious in their interaction with other people during radiation therapy with or without chemotherapy, this was another advantage.

Our reported infestation rate of 0.72% and the gerneral infestation rate of Munich with 0,43% also reflects the results of a larger Chinese study from Wuhan with 1524 oncological patients and an infestation rate of 0.73% [42, 43]. They also state, that this infestation rate was higher compared to all SARS-CoV-2 positive patients in Wuhan at the same time, showing the increased vulnerability of cancer patients.

Lung cancer patients were most susceptible, with 58% in the Chinese cohort and 100% (limited by sample size) in our cohort. This could be due to the fact that the lung is already being affected by the tumor and the frequently applied chemotherapy. Radiation can also lead to radiation pneumonitis in the lungs, which would probably cause even more fatal progressions in combination with COVID-19.

This strengthened our decision to postpone the therapy of this patient to avoid any deterioration caused by COVID-19 during radiotherapy. Although only one patient has been identified as an asymptomatic carrier based on the test performed so far, we will continue testing to make the best possible therapeutic decision for our patients and to provide the best possible safety for our staff.

A similar study conducted in the US by Ning et al. revealed a higher infestation rate of 5.8%. This higher infestation rate might result in their high percentage (55%) of symptomatic patients tested, whereas we only tested asymptomatic patients [44]. More interesting is the number of quarantined employees during that period. A total of 46 employees were quarantined as a precaution with a maximum of 17 at the same time. Such a high loss of employees at the same time would lead to a shutdown in smaller radiation departments underlining the importance of testing and the utilization of all preventive measures.

Since neither a therapy nor a vaccination to actively fight the virus is currently available, all these results are currently used to better assess the situation and identify potential conservative measures. With prevention as an important pillar of conservative measures, social distancing, higher hygiene standards, adaption of health care systems and lockdowns are currently under debate.

In these discussions, the base reproductive time (R_0_) plays a decisive role, indicating the transmissibility of a virus and representing the mean number of new infections generated by an infected person in a totally naïve population [45]. Because it is based on a virus-naïve population and the initial lack of sufficient test kits, R_0_ often varies especially with this newly emerging and rapidly spreading virus and is therefore discussed controversially.

Meanwhile with a far higher number of tests, all numbers including R_0_ are becoming more and more accurate and therefore more precise. Nevertheless, R_0_ is an estimate and can vary between days or regions tested. Zao et al. for example, reported that R_0_ was between 2.24 and 3.58, in contrast to an earlier report of their investigation where R0 was between 3.3 and 5.47. Others studies report a reproduction number of 1.3 up to 4.7, some even up to 6.47 [25]. This shows that different models and uncertainty in relation to the asymptomatic proportion in different testing populations leads to different results [46, 47]. A reported R_0_ value of < 1 in Germany in April 2020 showed that the prevention measures have generally fulfilled their purpose. With values now rising again and an uncertain outcome due to the easing of the lockdown in multiple countries, the continuation of testing is inevitable. While maintaining prevention measures, the expansion of testing capacities should now be encouraged in order to obtain a better picture of the real infestation rate of the population and asymptomatic patients.

Measured in terms of patient enquiries, the response was very positive, and thanks to the implemented measures mentioned above, we can carry out the radiotherapy services under optimal conditions with high hygiene standards. Patients welcomed the increased pandemic-adapted hygiene rules and procedures so far and the compliance was very high.

## Conclusion

A prerequisite for normalization to a pre-pandemic level would require an almost perfect drug for the treatment of COVID-19, or if almost every person on the planet would be vaccinated against coronavirus.

The option of a miracle drug that cures infected patients is probably not realistic in the foreseeable future. A vaccination may be available a little sooner, but it is not an option at the moment either.

So far, preventive measures are the only and most important option to fight the current SARS-CoV-2 pandemic regardless of whether all patients get tested or not.

Now, while maintaining all preventive measures, is the time to test more and more patients to derive objective numbers and prevent further spread. In our cohort of 139 asymptomatic cancer patients, the prevalence was 0.72%, which is in line with comparable studies. Current limitations are the time point and the quality of the one-time test. The limitation of the sensitivity of the PCR itself underlines the importance of preventive measures even if testing is done. Because even a single undetected patient in our department would be enough to further infect many others and risk the fragile success we have achieved so far with preventive measures.

## Supplementary information

**Additional file 1.** Document 1: Standardized Questionnaire.

**Additional file 2.** Document 2: Leaflet for outpatients.

**Additional file 3.** Document 3: Leaflet for inpatients

## Data Availability

All data generated or analysed during this study are included in this published article and its supplementary information files.

## Abbreviations

SARS-CoV-2: Severe acute respiratory syndrome coronavirus 2
COVID-19: Coronavirus disease 2019
SARS: Severe acute respiratory syndrome
MERS: Middle East respiratory syndrome
PCR: Polymerase chain reaction
WHO: World Health Organization
RKI: Robert Koch Institute, Germany
GCP: Good Clinical Practice
GEP: Good Epidemiological Practice
CT: Computed tomography
RTT: Radiotherapy technologist
R_0_: Base reproductive number
