# Supplementary material for: SARS-CoV-2 prevalence in an asymptomatic cancer cohort - results and consequences for clinical routine
Source: Radiat Oncol. 2020 Jul 9;15:165. doi: 10.1186/s13014-020-01609-0 (PMC7344028; doi:10.1186/s13014-020-01609-0)
Supplement: Supplementary file 1 — Additional file 1. Document 1: Standardized Questionnaire. [file 13014_2020_1609_MOESM1_ESM.pdf]

Document 1: Standardized Questionnaire

Patient's name: _________________________

Do you have fever?

[ ] no [ ] yes, since when: _________________________

Do you suffer from chills?

[ ] no [ ] yes, since when: _________________________

Do you have a cough?

[ ] no [ ] yes, since when: _________________________

Are you feeling exhausted or do you have flu-like symptoms?

[ ] no [ ] yes, since when: _________________________

Have you had contact with a patient who was diagnosed with coronavirus infection?

[ ] no [ ] yes, when: ___________________

With whom: ___________________________________

Have you been abroad in the previous 14 days?

[ ] no [ ] yes, when and where: ____

Have you been in another federal state in the previous 14 days?

[ ] no [ ] yes, when and where: ____

Does a relative of yours have fever?

[ ] no [ ] yes, when and where _____________________________

Have your relatives had contact with a patient with proven coronavirus infection?

[ ] no [ ] yes, when: ___________________ With whom: ____________________

Has one of your relatives been abroad in the previous 14 days?

[ ] no [ ] yes, when and where: _____________________________

Have you already been tested for a coronavirus infection or are you planning to do so in the near future?

[ ] no [ ] yes, when and what was the result: _____________________________
