# Supplementary material for: SARS-CoV-2 prevalence in an asymptomatic cancer cohort - results and consequences for clinical routine
Source: Radiat Oncol. 2020 Jul 9;15:165. doi: 10.1186/s13014-020-01609-0 (PMC7344028; doi:10.1186/s13014-020-01609-0)
Supplement: Supplementary file 2 — Additional file 2. Document 2: Leaflet for outpatients. [file 13014_2020_1609_MOESM2_ESM.pdf]

Document 2: Leaflet for outpatients

Information on behavior in the radiation department due to the Coronavirus/ COVID-19

Dear patients, dear relatives,
Patients with a reduced immune system, as it is frequently observed in cancer patients due to systemic therapies or radiation, can develop major problems up to fatal courses when infected with the SARS-CoV-2 /coronavirus. We would therefore like to avoid that an infection with a corona virus is introduced into our wards or outpatient areas. This serves both to protect the patients and our staff, who are all currently needed to maintain our treatment. Your honesty is required here. Please bear in mind that in the event of a corona virus infection introduced by you or your relatives, many patients on our ward are at risk of death. We therefore ask you to support the measures proposed by us.

General rules:

- Reduce the number of contacts with other people to an absolute minimum, i.e. household members who cannot be accommodated elsewhere or who are needed for support. Household members should preferably be people in good health. If they are ill, it is even more important to follow the recommendations.
- Contact with people outside your household should be avoided, e.g. postmen, delivery services, neighbors, friends, acquaintances. Have deliveries left in front of the house or apartment entrance, wear a face mask and keep a distance of 1.5 - 2 m from these people.

Therefore, please also note the following for the outpatient area:

1. Outpatient appointment (e.g. consultation, tumor follow-up)

- DO NOT bring an accompanying person to the outpatient appointments, not even to the waiting area. Only necessary legal guardians or translators are currently permitted, if sufficient German or English language skills are not available. Also taxi drivers or other drivers are not allowed to enter our departments, as long as no urgent medical help is needed.
- Employees of the Red Cross, Malteser International or comparable patient transport organizations as well as necessary accompanying persons of patients who are unable to walk are still allowed to enter.
- In any case, please disinfect your hands before entering our clinic. Appropriate disinfection stands are available at all entrances.

- As in the case of influenza and other acute respiratory tract infections, coughing and sneezing etiquette, good hand hygiene as well as distance to the ill (approx. 1.5 to 2 meters) also protect against transmission of the new coronavirus.
- If you have a fever or other signs of infection or you visited a so-called risk area, it should go without saying that you should not enter the radiation department without consulting us.
- Avoid any physical contact at any time with other patients or other persons outside your household such as friends, neighbors or acquaintances.
- Children and young adults should generally not attend the clinic as accompanying persons. In this population group, the risk is very high that SARS-CoV-2 can progress without clinical signs of infection and can therefore spread silently.
- Reduce the physical contact with clinic staff to the necessary extent (e.g. physical examination as part of treatment or blood sampling).

2. Regularly repeated outpatient appointments

- Please inform us in advance (Telephone: XXX) if you as a patient suffer from fever or general symptoms of an infection (scratching of the throat, coughing, chills). We also ask for information if one of your relatives shows these symptoms. We must then inquire very carefully whether you or your relatives have signs of a coronavirus infection. We will then decide how to proceed in each individual case. Your honest answer is required here. Please bear in mind that in the event of a coronavirus infection introduced by you or your relatives, many patients in our clinic may be at risk of death.
