# Supplementary material for: SARS-CoV-2 prevalence in an asymptomatic cancer cohort - results and consequences for clinical routine
Source: Radiat Oncol. 2020 Jul 9;15:165. doi: 10.1186/s13014-020-01609-0 (PMC7344028; doi:10.1186/s13014-020-01609-0)
Supplement: Supplementary file 3 — Additional file 3. Document 3: Leaflet for inpatients [file 13014_2020_1609_MOESM3_ESM.pdf]

Document 3: Leaflet for inpatients

Information on behavior in the radiation department due to the Coronavirus/ COVID-19

Dear relatives, dear patients,
Patients with a reduced immune system, as it is frequently observed in cancer patients due to systemic therapies or radiation, can develop major problems up to fatal courses when infected with the SARS-CoV-2 /coronavirus. We would therefore like to avoid that an infection with a corona virus is introduced into our wards or outpatient areas. This serves both to protect the patients and our staff, who are all currently needed to maintain our treatment. Your honesty is required here. Please bear in mind that in the event of a corona virus infection introduced by you or your relatives, many patients on our ward are at risk of death.
We therefore ask you to support the measures proposed by us.

1. Visit of relatives

- In order to avoid infections, there is currently a strict ban on visiting our wards. This serves your own protection as well as that of other patients on the ward and we ask you to strictly obey this.
- Visits by children or young adults should generally not be made. In this population group, the risk of coronavirus infection without clinical signs of infection is very high.
- If you have a fever or other signs of infection or you visited a so-called risk area, it should go without saying that you should not enter the radiation department without consulting us.
- Before entering the wards or the radiation rooms, we ask you to disinfect your hands sufficiently. Appropriate disinfection stands are available at all entrances.
- Avoid any unnecessary physical contact between you and your relatives even outside our wards.
- Please also make sure that other accompanying persons (driver, taxi driver etc.), if necessary, also disinfect their hands when entering our ward.

2. Regular admissions to the ward for inpatient admission.

- Please be sure to inform us in advance using the respective ward number below if you as a patient suffer from fever or general symptoms of an infection (scratching of the throat, coughing, chills).
- We would also ask you to inform us if one of your relatives has these symptoms. We will then decide how to proceed in the individual case. Your honest answer is required here. Please

bear in mind that in the event of a coronavirus infection introduced by you or your relatives, many patients on our ward are in mortal danger.

3. If you have fever, diarrhea, chills, nosebleeds, etc. during a treatment with us

- We urgently ask you to contact us by telephone first, so that we can rule out the suspicion of a coronavirus infection. We will of course continue to look after you as an inpatient if these symptoms indicate a tumor or therapy-specific cause. If there is a suspicion of a coronavirus infection, we will give you concrete advice on how to proceed.
